# Gross on Excision of the Maxillary Bone

**Published:** 1852-10

**Authors:** S. D. Gross

**Affiliations:** Professor of Surgery in the Medical Department of the University of Louisville


### *Observations on Excision of the Superior Maxillary Bone.*

By S. D. Gross, M. D., Professor of Surgery in the Medical Department of the University of Louisville.

The diseases of the superior maxillary bone, requiring the operation of excision, and generally described under the vague and unmeaning name of *osteo-sarcoma*, constitute a numerous and important group of affections, defying every attempt at correct classification. That this declaration is not a mere assumption, unsupported by facts, the writings of pathologists and surgeons abundantly attest. Much as has been written upon this subject within the last quarter of a century, there is still room for observation, both as it respects the nature, diagnosis, and treatment of these maladies. More light must be elicited before this branch of surgery can be placed upon a sure and solid foundation in relation to the propriety or impropriety of the operation of excision in any particular case, or in any particular class of diseases.

It is not my design, in this paper, to write a learned and elaborate article upon these affections; on the contrary, all I shall aim to do is, to point out a few of the more common and important, those which I have most frequently witnessed in practice, and those with which I am, consequently, most familiar from personal observation.

1. By far the most frequent disease of the upper jaw is *encephaloid*, osteocephaloma, or soft cancer, which occurs here, as elsewhere, in both sexes, in all classes of individuals, and at all periods of life. I have seen it in young children under five years, at middle life, in old age, and in decrepitude. I am satisfied, however, that is most frequent between the twentieth and fortieth years. In one of my patients it occurred at the age of sixty-six.

It is not known what influence, if any, is exerted upon the development of this by *occupation*, temperament, climate, and other circumstances. In every instance of it that has fallen under my observation, the tumor sprang up without any assignable, or palpable cause. Cases have been recorded in weich it was apparrently awakened by external injury, as a blow, the pressure of the pullikin, of fracture of the bone.

Encephaloid usually *begins* in the cavity of the antrum of Highmore, in connection with the mucous lining, or internal periosteum. Occasionally it takes its rise in the cancellated structure of the bone, in the socket of one of the teeth, in the gum, or in the periosteum, properly so called. In the first case, it generally progresses, with more or less rapidity, until it fills up the whole sinus, after which it enroaches upon the bony parietes of the cavity, pushing them out in every direction, and thereby pressing them against the surrounding structures. As the external wall is extremely thin, in fact, a mere shell, in the natural state, the morbid growth generally advances more rapidly in this direction than in any other, forming thus frequently, even at an early stage, quite a large tumor beneath the integuments of the cheek. By and by, as it proceeds in its development, it extends inwards towards the nostril, partially, sometimes wholly, occluding the corresponding cavity; upwards towards the floor of the orbit, compressing, and ultimately protruding the ball of the eye; downwards towards the roof of the mouth, displacing the tongue, and diminishing the buccal cavity; and backwards towards the fauces, impeding mastication, deglutition, speech, and respiration. At this stage of the disease, the countenance is most hideously disfigured, and the patient is an object well calculated to excite the deepest commisseration.

The integuments and mucous membrane are generally sound in the *earlier stages* of the malady; but after a certain period, varying from several months to a year or more, they begin to assume a livid hue, become deeply congested at one or more points, and at length yield to ulcerative action. The consequence is, a fungating and rapidly spreading sore, the seat of a thin, sanious, mucopurulent, or sanguinolent discharge, very abundant, excessively fœtid, and highly irritating in its character. Pure blood often proceeds from it; sometimes very small in quantity, at other times so copious as rapidly to undermine the strength, and bring on hectic irritation and exhausting night sweats.

In the *latter stages* of the disease, sometimes before, but generally not until after ulceration has set in, the lymphatic glands of the temple, behind the ear, and under the jaw become enlarged and contaminated, and finally bnrst from over distension of their contents. The countenance assumes a peculiar cadaverous hue and expression; the patient rapidly loses flesh and strength; colliquative diarrhœa and sweats supervene; the pain is excessive; and death, now a welcome visitor, closes the scene of agony and strife.

The *pain* attending this affection has no fixed or definite character. In general, it is dull and aching, not unlike the tooth-ache, to which, in fact, it is usually at first referred by the patient. As the tumor augments in volume, and enroaches upon the surrounding structures, the pain commonly becomes more severe and constant, and assumes a sharp, lancinating character. It is usually most violent at night, and in damp, cloudy states of the atmosphere; it is also greatly influenced by the condition of the stomach and bowels, by mental emotion, and by whatever has a tendency to produce derangement of the secretions. In some cases the pain is tensive and pulsatile, especially when the tumor is very large, or is about to become the seat of suppuration. Occasionally, again, it is of a neuralgic character, having, like an intermittent fever, regular paroxysms and intermissions. It is rarely limited to the diseased mass, but extends through the neighboring parts, and sometimes even to a considerable distance.

The *progress* of the tumor is variable, sometimes very rapid, at other times quite tardy. In 1844, a boy, five years of age, was brought to me for an affection of this kind, which caused his death in less than six months from its commencement. The tumor was of extraordinary volume, filling the mouth, throat, and nostrils, and causing the most frightful deformity. A short time before death, it gave way in the mouth, fungating and bleeding so profusely as rapidly to undermine the foundations of life.

A case of still more rapid termination was under my observation last summer, in a colored man, aged twenty-seven, belonging to a Mr. Lewell, of Palmyra, Tennessee. The disease, occupying the right maxillary bone, was first noticed in April, 1851, and by the 2d of July, when the man arrived in town, it had already attained an enormous bulk. The tumor formed a large prominence on the cheek, and almost filled the mouth, rendering it very difficult for the patient to swallow, and to articulate so as to make himself intelligible. It rapidly increased in size, extending behind the ear to the eye and temple, beyond the median line, and below the inferior maxillary bone, throwing the chin several inches over to the opposite side. In a few weeks after his arrival, a small slough formed upon the cheek, over the most prominent portion of the tumor, evidently from the effects of the pressure of the morbid mass, and this was soon followed by the appearance of a large fungus, which was the seat of a constant sannous and most loathsome discharge, attended, occasionally, with copious hemorrhage, and the most violent pain imaginable. Under the influence of this accumulation of evils, his general health already much impaired by previous suffering, rapidly gave way, and on the 9th of August he died. Upon inspection, the tumor was found to be composed of a soft, fluctuating, brain-like substance, the true character of which it was impossible to mistake.

In this case, no one could, of course, for a moment entertain the question of an operation; the period for such a procedure, if proper at any time, had already passed when the man reached Louisville; and the only reason why he was not sent back, at once, was that he was unable to bear the fatigues of the journey.

On the other hand, I have seen several cases in which the fatal event was delayed for several years. My experience is that the malady is usually more rapid here, as in other parts of tbe body, in children and youth than in the middle aged and old.

When such a tumor is *examined*, after removal, it is found to exhibit all the characteristic features of encephaloid formations. That portion which occupies the antrum is commonly quite soft and pulpy, resembling, at least faintly, a section of the brain, both in its color and consistence. The osseous structure is generally broken down and disorganized, quite vascular, and so soft as to be easily cut with the knife. In some specimens it is entirely, or nearly entirely absorbed; while, in others, it is replaced by fibro-cartilage, or cartilage, intermixed with spicula and scales, remnants of the original substance. In nearly all cases, the morbid mass is remarkably vascular, being pervaded, in every direction, by large vessels, the walls of which are exceedingly brittle, and therefore liable to yield under the slightest impulse. It is owing to this circumstance that these tumors frequently attain such an enormous volume, and that, when ulceration sets in, they are so liable to fungate and bleed.

The *recognition* of encephaloid disease of the superior jaw is usually not difficult. The rapid growth of the tumor, its steady encroachment upon the adjacent parts, its soft and elastic feel, the livid aspect of its buccal portion, and the sharp darting pains which attend it, readily distinguish it from all other formations of this division of the skeleton. In the latter stages of the affection, the fungous character of the ulcer, and the sanious, sanguinolent, and bloody discharges, together with the sallow and cadaverous state of the countenance, and the enlargement of the neighboring lymphatic ganglions, leave no doubt about its nature. Important information will also be furnished by the history of the case, and by the fact that encephaloid occurs at all periods of life, while some of the other maladies of this region are met with only at certain ages.

Where any doubt exists respecting the character of the tumor, no objection lies against the use of the exploring needle, which will at once inform us as to the consistence of the tumor, and the nature of its contents. If it is encysted in its character, an escape of serous, or muco-sanguineous fluid, will afford the necessary intelligence, and enable us to arrive at a pretty satisfactory conclusion in regard to the proper mode of procedure. Should encephaloid matter be present, the smallest quantity, in fact the merest particle, will, if subjected to the microscope, reveal the characteristic cancer-cell. I consider it of great importance that the exploring instrument should be as fine as possible, that the puncture which it makes may heal by the first intention, and not permit the protrusion of any of the contents of the tumor. There is much danger in malignant diseases, where this precaution is omitted, of doing harm by inducing premature ulceration, fungation, and hemorrhage, followed by the rapid destruction of the patient. I much fear that surgeons have paid too little attention to this circumstance.

Encephaloid of the jaw seldom *co-exists* with malignant disease in other parts of the body. The affection, in fact, in the great majority of instances, is more habitually local than when it invades the cellular tissue, eye, and glandular organs. It is, doubtless, owing to this circumstance that excision of the disease, especially in its early stage, is occasionally followed by a successful result; though, in general, as was before intimated, the prognosis is most unfavorable.

2.—The tumor next in point of frequency is the *fibro-cartilaginous*, or, as is it not uncommonly, but incorrectly termed, *sarcomatous*. It is not easy to describe this variety of growth, so diversified and multiform are its component elements. Its most ordinary form is the fibrous, in which, as the name implies, the fibrous structure predominates, though there is often, if, indeed, not constantly, an intermixture of other elements, especially the cartilaginous; small cysts, containing a thin serous, glairy, or sanguineous fluid, are sometimes interspersed through these tumors. Few vessels are apparent in their structure, and hence they seldom attain a very great bulk, or advance with much rapidity. For the same reason they do not bleed much when ulceration takes place, or during attempts at extirpation.

The original site of this tumor, like that of encephaloid, is usually the antrum; but it also sometimes begins in the proper substance of the bone, in the socket of one of the teeth, usually in that of one of the grinders, in the periosteum, or between the periosteum and the outer surface of the bone. However this may be, the substance of the bone is gradually broken down and disintegrated, and ultimately lost in the new deposit.

The fibro-cartilaginous tumor of the upper jaw, notwithstanding that it is not, in general, very vascular, occasionally attains a very large size. Instances have been reported in which it was as big as a fœtal head. Usually, however, it is small, not exceeding the volume of an orange, or an ordinary fist. One of the most interesting facts connected with this tumor, is its non-malignant character, or its tendency not to return after extirpation.

The fibro-cartilaginous tumor can generally be *distinguished* from encephaloid and other formations of the upper jaw, first, by the slowness of its growth; seccondly, by its globular, ovoidal, or botryoidal shape; thirdly, by its circumscribed character, or indisposition to ramify through the surrounding parts; fourthly, by its firm and unyielding feel; fifthly, by its painlessness; and sixthly, by the absence of contamination of the neighboring lymphatic glands. The tumor manifests little or no tendency to ulcerate, and the mucous-membrane of the mouth is of a more florid color than in encephaloid. There is also less serous and bloody discharge. The general health is not deteriorated, and the countenance is free from the peculiar sallow and dejected expression which forms so remarkable and characteristic a feature in malignant disease. The fibro-cartilaginous tumor is not confined to any particular period of life, but is most common in young adults.

3.—The *epuliform* tumor is occasionally seen upon the upper jaw, though its favorite site is the lower. This is a sarcomatous, or fibrous growth, of a soft spongoid consistence, and of a red florid complexion, springing originally from the gum, or the lining membrane of the socket of a tooth, and gradually extending to the bony texture, which, in time, is partially absorbed, or expanded into a soft, elastic, parchment-like shell. By and by, the teeth loosen and drop out; the morbid mass ulcerates and fungates; and the patient is constantly annoyed by a sanious, or bloody discharge, so offensive as to be loathsome both to himself and to his neighbors. The tumor seldom extends beyond the alveolar process; but occasionally it forces its way, even at an early period, into the maxillary sinus, rapidly filling it up, and then protruding its walls in every direction, as in the true osteocephalomatous formation. A decayed tooth, a blow upon the jaw, or injury upon the gum, are the most common causes of this affection. Not unfrequently, however, it comes on spontaneously, under the influence, apparently, of some constitutional taint.

The *diagnosis* of this form of tumor rarely offers any difficulty. Its exposed situation, its connection with the teeth and alveolar process, its want of sensibility, its florid appearance and spongoid consistence, are almost pathognomoniac of the nature of the affection. It is only in the more advanced stages of the malady, when the body of the jaw has become extensively involved, when the surface of the tumor has begun to ulcerate and sprout, and the discharge is copious, bloody, and offensive, that the discrimination will be likely to prove embarrassing. The tumor, under such circumstances, might be easily mistaken for medullary sarcoma; a circumstance, however, which need not be particularly regretted, inasmuch as the curative measures are the same in both cases.

The *progress* of this morbid growth is sometimes remarkably rapid; at other times, slow and gradual. As it increases in volume, it must necssarily encroach very materially upon the mouth, thereby interfering with the tongue, teeth, and fauces. Its character is usually malignant, and hence, unless it is thoroughly extirpated, it is almost certain to return after removal. Like encephaloid, it rarely co-exists with malignant disease in other parts of the body. Indeed, it would seem, even in its worst forms, to partake more of the cancroid than of the true cancerous structure and habit.

4.—The *sero-cystic* tumor of the upper jaw is uncommon, and, fortunately, devoid of malignancy. Its usual site is the alveolar process, where it has been known to attain the volume of a hen's egg, and even of a large orange. It is composed of a thin, semi-transparent, or slightly opake bag, occupied by a colorless, or sanguineous fluid, or by a glairy, mucilaginous substance. Sometimes, though rarely, there are two such cysts, either closely connected together, or separated by a kind of osseous septum. The bone around the tumor is expanded into a thin, elastic, crackling, parchment-like shell, and is easily penetrated by a sharp instrument, the puncture giving vent to the characteristic contents of the cyst. This, in fact, is the best diagnostic sign of the morbid growth. The general health remains unaffected in this disease, which is always tardy in its progress, and manifests no disposition to extend among the adjacent structures. Where any doubt exists as to the real nature of the case, a resort to the exploring needle will usually at once dispel it.

It is not often that this tumor calls for removal of the affected bone; in general, it will suffice to puncture it occasionally with a small trocar to evacuate its contents, the escape of which is often followed by the rapid contraction and final obliteration of the sac. Where there is a strong tendency to re-accumulation, a larger opening should be made, and tincture of iodine thrown in, to promote the object in view. It is only in old and intractable cases that excision of the affected bone will be likely to be necessary.

5.—Excision of the superior maxillary bone is sometimes required on account of the presence of an *exostosis*. The tumor, varying infinitely in regard to its size and form, is most common in young and middle aged subjects; it may appear upon any part of the bone, and gradually augmenting in volume, may at length involve it in its entire extent. It is a strictly local affection, the result often of external violence; and rarely, if ever, degenerates into malignant action. An exostosis is a true bone, growing, as the name implies, from bone, and composed of precisely the same anatomical, chemical, and microscopical characters.

An exostosis is easily *recognized*. Its chief peculiarities are, its excessive hardness, its slow growth, its freedom from pain, the absence of disease in the surrounding structures, and the unimpaired state of the general health. There is no discharge of blood, or muco-purulent matter, no tendency to ulceration, no alteration in the skin of the face, or mucous membrane; the principal inconvenience which the patient experiences is from the size of the morbid growth, which is occasionally very considerable, and from its consequent interference with the functions of the adjacent parts. When doubt exists in regard to the real character of the disease, a small exploring needle, introduced at various points of the swelling, will at once decide the question.

6. Under the same head with exostosis may be mentioned an enlarged state of the bone, constituting a species of *hypertrophy*. An example of this variety of morbid action will be found in the first case appended to this paper, in which the tumor, occurring in a young lady of nineteen years, was occasioned by the irritation of an inverted, or misplaced canine tooth. The bone was expanded into a thin shell, about the volume of a large hickory nut, and was of a hard, firm consistence, with but little pain, and no constitutional derangement. The tumor was evidently of local origin, and the operation, performed in April, 1845, was perfectly successful; the part remaining well up to the present moment.

Mr. Stanley* mentions an instance in which the hypertrophy involved the whole jaw, which was removed by operation; the patient, however, a girl of fifteen years, dying on the tenth day from erysipelas. The cavity of the antrum was completely obliterated by the osseous matter, which exhibited all the characters of the natural texture. The general health was perfectly good, and no disease was discoverable in the surrounding parts. The hypertrophy had been in progress for eight years, and presented itself mainly in an oblong, hard, and painless enlargement of the ascending portion of the bone, projecting into the face, and extending into the nostril.

Such enlargements, it is obvious, are not malignant, and are, in general, easily distinguished from this class of affections by their slow developement, their firm, unyielding consistence, their painless character, and the healthy condition of the adjacent parts.

7.—*Scirrhus* of the superior jaw must be exceedingly rare, as I have not, in a practice of nearly twenty-five years, met with a solitary case. Many instances of the kind are, I am aware, reported in our surgical and periodical literature; but

*Treatise on the diseases of the bones, p. 227; Phil. 1849.

it is perfectly clear to my mind that they were examples, not of scirrhus, but of encephaloid. Should this disease ever occur here, it would be likely to show itself in advanced life, as a hard, firm, solid tumor, slow in its progress, and characterized by sharp, lancinating pain. The growth would be likely to attain much less bulk than soft cancer; nor would it be so liable to fungate and bleed.

8.—*Melanosis* of the upper jaw is likewise very unfrequent, and no example of it has yet fallen under my observation. The same remark is applicable to colloid, which is, if possible, a still more rare disease than melanosis. A fatty tumor has been described as occurring in the upper jaw; and Gensoul gives an instance wherein the antrim was occupied by an erectile growth. The tumor, in this case, was remarkably soft, and so vascular that it appeared, after having been injected with quick-silver, to be composed chiefly of minute vessels.

9. *Polypoid* tumors of the antrum are occasionally seen, but much less frequently than is generally supposed. Indeed, they must be exceedingly rare. They are benign or malignant, generally the latter; are difficult to diagnosticate; and are always prone to return after extirpation. These tumors require no particular description; because they do not, in the great majority of instances, differ from the medullary formation, from which, in fact, it is usually impossible to distinguish them.

It would seem that *exsection* of the upper jaw was practised by Acoluthus as early as 1693. The operation was repeated, in the eigteenth century, by Planque, David, and Beaupreau, and, early in the present century, by Siebold, Deschamps and Klein.* In all these instances, however, it was limited to the removal of a portion of the bone. In Great Britain, extirpation of the upper jaw does not appear to have been practised until 1826, when it was executed by Mr. Lizars, of Edinburg, who, I believe, is fully entitled to the merit of

*Velpeau's Operative Surgery, 2. p. 728.

having first suggested the possibility and advantage of removing the entire bone, when the seat of disease.* Three years prior to this period our countryman, Dr. Alexander H. Stevens, then Professor of Surgery in the University of New York excised nearly the whole of the upper jaw, along with portions of the malar, ethmoid, and sphenoid bones, for a fungus tumor of the antrum.† In May, 1824, Dr. David L. Rogers, of New York, performed a similar but still more formidable operation, exsecting the superior maxillary bones, anterior to the first molar tooth on each side, together with the two inferior turbinated bones, a part of the nasal septum, the vomer, and a portion of the right antrum.‡ Since the period here adverted to, the operation in question has been repeatedly executed by American surgeons, among whom McClellan, Mott, Mussy, Mutter, Pancoast, and the two Warrens, deserve special mention. I am not aware that it has been performed by any one in Kentucky, except myself.

Previously to undertaking any operation for the removal of the upper jaw, whether for a part or the whole of it, a careful inquiry should be instituted into the history of every case, with a view of ascertaining its particular character, the condition of the adjacent parts, and, above all, the state of the general system. If the tumor be of the cephalomatous kind, large in size, rapid in its development, dipping into the surrounding structures, and attended with contamination of the neighboring absorbent glands, failure of the general health, and a sallow cadaverous countenance, all manual interference is out of the question. To operate, under such circumstances, would only hasten the patient's fate, and tend to bring surgery into discredit. On the other hand, however there is no reason, in my opinion, for refraining from the use of the knife, even in this form of disease, provided the morbid growth is comparatively small and tardy in its pro-

---

*Liston's papers on Tumors of the Mouth, in London Medico-Chirur. Trans. 20. p. 178.

† Cooper's Surgical Dictionary, appendix by Dr. Reese.

‡Surgical Essays, p. 81, New York, 1848.

gress, and the surrounding parts and constitution are perfectly sound. It is a well ascertained fact, as was previously stated, that encephaloid of the upper jaw rarely co-exists with malignant disease in other organs; and, hence, if such a tumor be exsected at an early period of its development while its action is strictly local, success must occasionally crown our efforts, at least so far as to prolong the patient's life, if nothing more, beyond the period he could otherwise attain. The propriety of such a course is fully sanctioned by the experience of the best of surgeons. In the case of Mrs. Green, the third on my list, although the disease for which the bone was removed was unquestionably of an encephaloid nature, and has repeatedly returned, five years have elapsed since the first operation, but for which she must have perished long ago. The great fault, in regard to the operation, is, that it is generally not resorted to sufficiently early in the disease, owing to the ignorance of the patient, and his unwillingness to submit to the knife as long as he can persuade himself that he may be relieved by ordinary means. The case being thus trifled with, the surgeon, when it falls into his hands, finds himself in the disagreeable dilemma of being obliged to decline all interference, or to attempt an operation, the result of which is almost sure to be unsatisfactory. He has to contend with science and self-interest, on the one hand, and sympathy for his patient, on the other; and his decision will be influenced by the predominance of the one or the other. Most men, if not wholly governed by selfish considerations, would be likely, if the case is not altogether desperate, to afford the sufferer a chance for his life. I do not wish to be understood, in making this remark, that I am an advocate for the indiscriminate use of the knife; by no means; on the contrary, as is well known to my friends and pupils, no one more constantly and loudly condemns a reckless and unprincipled resort to instruments. I merely wish to say, and I say it boldly, that I abhor timid surgery, and that cautious, calculating conduct in operators which induces them to pay more regard to their own reputation than to the welfare of their patient; operators who would not hesitate to consign the poor sufferer to his cruel fate, because they are afraid of public opinion. I do not envy such men their feelings; I despise them as much, and as cordially, as I despise the merest knivesmen. It should never be forgotten that a case, which may appear hopeless in the eyes of one surgeon, may not appear so in the eyes of another, equally enlightened; and that a case, which one surgeon has condemned as unsuitable for an operation, has sometimes been easily relieved by another equally expert. It cannot be denied that surgery has achieved some of its proudest triumphs in this manner.

In non-malignant tumors of the upper jaw, no fears need be entertained about a relapse; the only difficulty here is about the diagnosis. When this has been clearly established, and the patient is in a proper condition for an operation, the sooner it is performed the better.

The *manner* in which the operation is to be performed, must necessarily vary with the circumstances of each individual case. When the tumor is of considerable size, requiring sometime for its removal, the patient should always be placed recumbent, with a broad and rather thin pillow under the head and shoulders, and the face inclining to the opposite side. Very few persons, whatever may be their courage and fortitude, can bear the shock and fatigue of an operation of this kind while sitting up. The recumbent position is more particularly necessary when chloroform is administered. I find considerable objection expressed by certain gentlemen against the use of this article in operations upon the mouth; but without, it seems to me, any just reason; at any rate, I have resorted to this agent ever since its introduction into the materia medica in every severe case of amputation, both of the upper and lower jaw, that has come under my observation, and I have, thus far, had no cause to regret the practice. The only objection that can be urged against anæsthetics in these operations is, that the patient being unconscious, is unable to clear his mouth of blood; but if proper care be taken to compress the vessels as soon as they are divided, and the head be placed upon a low pillow, no danger can ensue from this source; I certainly have never witnessed any, and until I do I shall not hesitate to continue the practice, whatever others may say and do.

I have never found it necessary, in any of my operations upon the upper and lower jaw, to secure the carotid artery, as a means of preventing hemorrhage. Indeed, one cannot but be surprised that such a precedure should ever have been recommended, much less employed, by any sensible surgeon. My experience is that there are no organs in the body, of the same extent in their natural and diseased condition, the removal of which is attended with so little hemorrhage. I am not in the habit even of employing compression of the carotid artery in these operations; and, as to tying that vessel as a means of security against the loss of blood, I should as soon think of ligating the femoral artery for the same purpose. Nothing could be more absurd and unnecessary. The chief danger from bleeding is in the subcutaneous arteries, especially the facial and its branches, and expert assistants should always be at hand to seize and compress them as soon as they are divided. I rarely, if ever, stop to tie a vessel during any operation, however extensive, or complicated. My experience has taught me that there is, in general, no necessity for such a course, which is always attended with vexatious delay and annoyance. The deep seated arteries, involved in tumors of the upper jaw, seldom bleed much, if care be taken to keep beyond the limits of the diseased structures. If this precaution be neglected, the hemorrhage may be copious, and even exhausting. The oozing which takes place from the osseous surface, after exsection has been effected, generally speedily ceases of its own accord from the contact of the atmosphere; where this is not the case, a stop may usually be easily put to it by the application of compresses, wet with a saturated solution of alum. The actual cautery could be necessary, in such a case only, when a portion of the tumor has been left behind; a circumstance which ought never to happen in the hands of any one, as it must necessarily lead to a speedy reproduction of the mischief.

The direction, extent, and number of the *incisions* through the soft parts must, of necessity, vary with the situation and extent of the tumor. In all these respects, much must be left, in each case, to the judgment and experience of the operator. When the morbid growth is comparatively limited, and seated upon the anterior, or antero-lateral aspect of the jaw, we shall generally be able to dispense with external incisions altogether, as our object may be readily enough accomplished simply by dissecting off the lip from its attachments, and holding it out of the way with the finger, or blunt hook. The surface of the tumor having been thus thoroughly denuded, the bone is to be attacked with the saw, and severed fairly beyond the line of disease. By this procedure, which is admirably adapted to the more simple forms of morbid growth, the operation is deprived of much of its severity, and followed by no deformity of the features, except what may result from the caving in of the integuments.

When the tumor involves the body of the bone, and is of considerable bulk, as, for example, as big as a fist, the plan which I usually adopt, and which has always answered my purpose most fully, is to make one long, curvilinear incision, extending across the most prominent part of the tumor, from the commissure of the lips towards the zygomatic process of the malar bone, terminating within a few lines, half an inch, or an inch of the external angle of the eye, according to the volume of the abnormal mass. In this manner are formed two flaps, the upper of which is convex, and the lower concave, which are then carefully dissected up by bold and rapid strokes of the knife, and held out of the way by trustworthy assistants, who at the same time take care to compress the bleeding vessels. The space which this procedure affords is, in general, quite sufficient for the easy removal of the entire tumor, however large, or extensive its connections. In my own cases, it has always answered my purpose most thoroughly. Should it, however, be inadequate, it can be easily increased to the requisite extent by a horizontal cut along the inferior border of the orbit, as far as the nose. In making the first of these incisions, the facial artery is necessarily divided, and in the second, the superior maxillary nerve, together with many of the branches of the portio dura of the seventh pair. In consequence of the injury thus sustained, the parts supplied by these nerves remain a long time paralyzed, though ultimately the face regains, in a great degree, its accustomed power and expression.

[TO BE CONTINUED.]

